# Enhancing PV power forecasting through feature selection and artificial neural networks: a case study

**DOI:** 10.1038/s41598-025-07038-x

**Published:** 2025-07-02

**Authors:** Mokhtar Ali, Abdelhalim Rabehi, Abdelkerim Souahlia, Mawloud Guermoui, Ali Teta, Imad Eddine Tibermacine, Abdelaziz Rabehi, Mohamed Benghanem, Takele Ferede Agajie

**Affiliations:** 1 Telecommunications and Smart Systems Laboratory, University of Djelfa, P.O. Box 3117, 17000 Djelfa, Algeria; 2 Unité de Recherche Appliquée en Energies Renouvelables, URAER, Centre de Développement des Energies Renouvelables, CDER, 47133 Ghardaïa, Algeria; 3 Department of Computer, Control, and Management Engineering, Sapienza University of Rome, 00185 Rome, Italy; 4 Physics Department, Faculty of Science, Islamic University of Madinah, 42351 Madinah, Saudi Arabia; 5 Department of Electrical and Computer Engineering, Institute of Technology, Debre Markos University, P.O. Box 269, Debre Markos, Ethiopia

**Keywords:** PV power, Renewable energy, Features selection, Forecasting, Artificial neural networks, Energy science and technology, Engineering, Mathematics and computing, Physics

## Abstract

This paper presents a comprehensive investigation into enhancing photovoltaic (PV) power forecasting by systematically integrating feature selection techniques with artificial neural networks. Addressing the growing demand for reliable renewable energy forecasting, the study employs several feature selection methods, including ReliefF, minimum correlation, Chi-square test, and others, to identify the most relevant predictors for PV output prediction. Two predictive models, the multilayer perceptron (MLP) and long short-term memory (LSTM) networks, are developed and tested on a real-world dataset from southern Algeria. The results demonstrate that applying feature selection significantly improves forecasting accuracy. For instance, integrating ReliefF with MLP reduced the normalized mean absolute error (nMAE) to 9.21% with an R^2^ of 0.9608, while the best LSTM configuration achieved an nMAE of 9.29% and an R^2^ of 0.946 when using Chi-square selected features. These findings confirm that careful feature selection enhances model performance, reduces complexity, and ensures better generalization, offering valuable insights for more efficient solar energy management and grid stability.

## Introduction

**Fig. 1:**
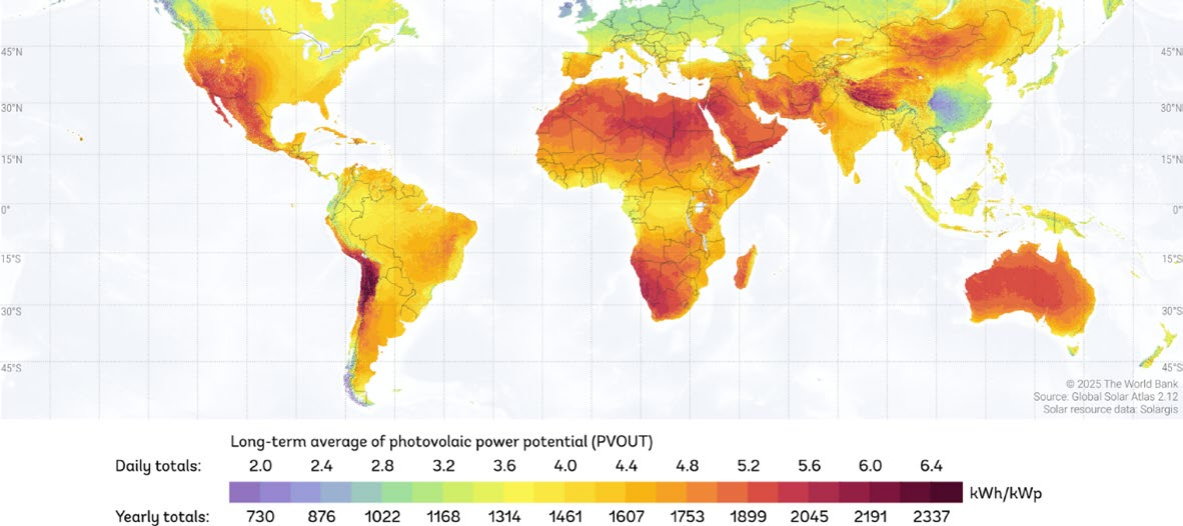
Practical photovoltaic power potential at level 1 (long-term average), Source: Global Solar Atlas, © 2025 The World Bank; Solar resource data: Solargis. Available at^2^.

Solar energy is clean and abundantly available. Solar technologies utilize the sun to provide light, heat, electricity, etc. for domestic and industrial applications. With the alarming rate of depletion of major conventional energy resources such as coal, oil, and natural gas, coupled with the environmental degradation caused by the process of exploiting these energy sources, it has become urgent to invest in renewable energy resources that would sufficiently power us in the future without degrading the environment through greenhouse gas emissions. The solar energy potential is immense, but despite this unlimited solar energy resource, its harvesting is challenging, mainly due to the limited efficiency of photovoltaic cells. The demand for renewable electricity sources is rapidly increasing due to global efforts to reduce CO_2_ emissions^1^. Photovoltaic energy, in particular, plays a promising role for both developed and developing countries and is considered the most promising renewable energy source due to its advantages. Firstly, photovoltaic energy is clean, as it can produce electricity without emitting greenhouse gases and toxic gases such as CO_2_ and NOx. Additionally, it can have positive economic effects, not only because it reduces electricity bills after the initial investment, but also because the renewable energy sector has the potential to create new jobs. Furthermore, technologies harnessing solar energy are relatively easy to install on rooftops and can thus provide a means of producing clean electricity in rural areas. The potential for electricity production from photovoltaic converters essentially depends on the region where they are installed. Specifically, the production potential depends on average solar irradiation as shown in Fig. 1^2^. The numbers indicated on the map presented in Fig. 1 show that solar energy provides a great production potential. However, the best conversion efficiency of most commercially available solar cells is in the range of 10–20%^3^. Although recent breakthroughs in solar cell technology show significant improvement, the fact that the maximum efficiency of solar cells still lies within the range of less than 20% indicates that there is a huge room for improvement. Algeria is indeed one of the largest solar energy deposits in the world with a duration of sunshine from 2000 to 3900 h per year, and a daily irradiation of 3000 to 6000 Wh/m^2^, equivalent to 10 times the global consumption which gives a great possibility of exploitation in this energy.

The problems related to the integration of photovoltaic production into an electrical grid are becoming increasingly concerning, thus requiring the implementation of adequate solutions to mitigate the impact of these productions on the grid^4^. Among the main solutions envisaged are the development of forecasting and storage tools, thereby allowing the grid manager to anticipate and/or guarantee the level of production connected to the electrical grid. To address these challenges, the grid manager must anticipate variations in solar energy production and other intermittent sources over several time scales. This includes medium-term forecasting (j + 1, j + 2, j + 3), which allows for long-term energy supply planning and anticipation of demand fluctuations. In the short term (h + 1, h + 24), forecasting focuses on narrower intervals, facilitating real-time grid management and immediate decision-making to maintain the balance between supply and demand. Finally, in the very short term (m + 5), forecasting is done almost in real time, allowing for rapid response to sudden fluctuations in production or electricity consumption, including activation of available production means immediately. Thus, the development of forecasting and storage tools is essential to accompany these anticipations and ensure grid stability^5^.

The increasing use of artificial intelligence (AI) techniques in the field of renewable energies is driven by the recognition of its potential. Many problems associated with renewable energies correspond precisely to the types of problems for which the AI approach seems most suitable. These computational models aim to simulate the cognitive and sensory functions of the human brain, exploiting this capacity to represent and manipulate knowledge in the form of models. Artificial neural networks (ANN), for example, use these models to model input–output functional relationships and can thus make predictions on other combinations of invisible inputs. AI techniques offer the potential for more accurate, faster, and more practical predictions than traditional methods^6^. Moreover, data from photovoltaic energy systems, being inherently noisy, are ideal candidates for processing by AI systems.

Wan et al.^7^ presented an in-depth review of solar energy forecasting methods and their applications in the field of power grids. They classified these methods into four families of approaches, a statistical approach, an artificial intelligence (AI) approach, a physical approach, and a hybrid approach. Statistical approaches are based on statistical techniques in the field of time series analysis using historical data, such as the autoregressive integrated moving average (ARIMA) method. AI approaches use the latest advances in the field of machine learning such as artificial neural networks (ANNs), decision tree forests (RF), support vector machines (SVM), etc. This category of methods can also be classified in the family of statistical approaches. Physical methods are part of the field of numerical weather prediction (NWP) using data from weather forecasts and satellite images. Hybrid approaches combine approaches from the three families of methods. The choice of a forecast category results from several factors such as the available data, the forecast time horizon, the domain of use of the forecast, etc.

These applications are based on the forecast time horizon. Very short-term forecasting and short-term forecasting are used primarily in managing the operating activities of PV power plants, the automatic regulation of the production, storage control, and the energy market, etc. Medium and long-term forecasts are mainly used to manage network works and to maintain, evaluate and plan PV park installations in the grid. Antonanzas et al.^8^ presented an in-depth, comprehensive review of the latest advances in solar prediction research. They studied several articles to gather a large part of the knowledge on PV production forecasting and to identify the latest trends in the field. These works are distinguished by the time horizon ranging from a second to weeks and spatial horizons from a single site to a region. First, they introduced the purpose and different motivations behind solar forecasting. Second, they presented a summary of the different methods and techniques used in this work. Third, they presented the differences between point and probabilistic forecasts and the advantages of each category of the forecast. Then, they classified and summarized the different articles according to time horizons and input data. Finally, the metrics used in the forecast performance measure are presented. Van der Meer et al.^9^ presented a summary of recent work in the field of probabilistic solar power and load forecasting. The objective of this probabilistic forecast is to provide a complete predictive distribution of a future state or forecast that the forecast quantity will fall within a forecast confidence interval. They analyzed several works in this field and the performance of each method used. They concluded that no one model applies to all use situations. A model that performs well in one dataset does not necessarily perform well in other datasets. Their study aimed to find a link between the probabilistic forecast of solar production and the load to predict the actual net load consumed. They showed that these two types of forecasts are similar in terms of spatial and temporal resolution but that the similarity in terms of variability between production and load can differ significantly.

Hong et al.^10^ organized an energy forecasting competition entitled “the Global Energy Forecasting Competition 2014” (GEFCom2014) In this competition, participants developed probabilistic forecasting approaches for the charge, energy price, wind energy, and solar energy. This competition attracted 581 participants from 61 different countries. The advantage of this type of work is the standardization of the metric and the data used. The participants worked on the same datasets, and the same metric evaluated their models. The organizers used data from three PV stations in Australia with a granularity of 1 h and forecast data from 12 meteorological variables in the solar forecast part. The objective of this competition was to provide a forecast of the solar production of these three PV parks over a 24-h horizon. Predictions had to be expressed as 99 quantiles. The top five winning teams used algorithms from the statistics and machine learning category like gradient boosting (GB), kNearest Neighbor (k-NN), quantile regression forest (QRF), multiple quantile regression (MQR), RF, and SVM. The winning team used GB and k-NN using all the information provided. Russo et al.^11^ presented the problem of solar energy forecasting and state-of-the-art forecasting methods. Then, they presented their short-term forecasting approach of 1 h ahead forecasting using several input data. Using a genetic algorithm, they leveraged data modeling software called “The Brain Project”. The forecasting approach is evaluated on data from a PV park with a capacity of 1.05 kW located in Catania, Italy. They used multiple input data. The results showed that algorithms with only two inputs perform better than naïf algorithms.

Rana et al.^12^ used ML methods for short-term forecasting of PV energy production by exploiting production curves and meteorological data. They applied the ANN and SVM algorithms with two different learning methodologies. One approach only uses production data (endogenous data). This approach is called the univariate approach. A second approach is a multivariate approach that uses endogenous and external meteorological data (exogenous data). The results showed that the univariate approach is more accurate in forecasting quality than the multivariate approach, with a mean relative error (MAPE) between 4.15 and 9.34%.

Lin et al.^13^ applied two ML algorithms (ANN and SVR) to forecast the energy of a 6 MW solar park in Florida, USA, over multiple time horizons (15 min, 1 h, and 24 h). They evaluated their approach using endogenous and exogenous (weather) data. The endogenous data is made up of the measurement histories of 11 inverters in the PV fleet. They developed a hierarchical forecasting approach using data from these inverters. That is, they created a forecast model for each inverter. Subsequently, the inverter forecasts are aggregated at the PV park level. They showed that the inverter forecasts improve the forecasting quality of the PV systems compared to conventional approaches.

Golestaneh et al.^14^ presented a methodology for probabilistic forecasting of solar production. They used an extreme learning machine (ELM), which provided point-in-time and probabilistic solar power predictions ranging from minutes to an hour. They evaluated different forecasting approaches on several production data in regions with different climates.

Bessa et al.^15^ developed a probabilistic forecasting algorithm for PV production over a 6-h horizon. They used several PV production curves collected in an electricity distribution network in the city of Évora in Portugal. Their results showed that combining all these data in a common forecasting framework can improve the forecast’s accuracy (between 8 and 12%) compared to a univariate model.

Hosain et al.^16^ developed an approach for forecasting solar production over a 1-h and 1-day horizon using the ELM algorithm. They evaluated their model using endogenous historical data from three PV parks in Malaysia and exogenous meteorological data. Their results showed, by comparing with other algorithms (SVR and ANN), that the ELM algorithm provides better forecast quality in a reduced computation time.

Lonij et al.^17^ developed a solar prediction approach under cloudy skies for 80 rooftop PV arrays spread over a 50 km × 50 km area in Tucson. They used data from several sensors installed in the study area with a granularity of 15 min. Their forecasting approach using correlations between photovoltaic system production was more accurate in forecasting compared to the naïf models and approaches using satellite image data.

Vaz et al.^18^ developed a forecasting approach for up to 1 month using the NARX algorithm (Nonlinear Autoregressive with exogenous inputs). They evaluated their methodology on weather data and historical data from nearby PV farms. They showed that the use of historical data from neighboring parks improves the forecasting quality for both winter and summer seasons. Their results showed that the NARX algorithm performed better than the naïf model for forecast horizons longer than 15 min.

Lin and Pai^13^ developed a monthly solar production forecast model in Taiwan using only historical production data. The authors have experimented with statistical techniques that can be used in the field of time series. They initially applied the technique of seasonal decomposition (SD) to remove the effect of seasonality in the production curve. Then, they elaborated the prediction using the least square support vector regression (LS-SVR) algorithm by optimizing the parameters by the genetic algorithm. The results showed that this algorithm is the most accurate in prediction with a MAPE of 7.84% compared with the other algorithms like ARIMA, SARIMA, ANN, and LSSVR without SD. They showed the importance of using SD in the data before developing the forecast.

Selecting appropriate predictors for effective PV power forecasting can be a time-consuming and challenging task. Randomly choosing predictors may lead to redundancy and correlation among data features, thereby increasing input dimensionality and complexity while decreasing forecasting performance. Thus, our work aims to identify optimal input features that reduce complexity and enhance forecasting performance. To achieve this goal, we propose employing a set of feature selection techniques to evaluate the relevance of commonly used predictors. Subsequently, an artificial neural network (ANN) is utilized to model measured daily global solar radiation using the identified optimal predictors. To assess the effectiveness of our approach, we employ a real dataset collected in the Ghardaïa area of Algeria from 2018 to 2019, and compute a set of objective evaluation metrics. The results yield a ranking score for each predictor, indicating its relevance. Consequently, the most relevant predictors are selected to train a multi-layer perceptron (MLP) model to predict measured PV power.

This paper proposes a systematic framework that integrates multiple feature selection methods, such as ReliefF and mRMR, with classical machine learning models to enhance forecasting accuracy. In particular, we employ and compare two neural network architectures, the multilayer perceptron (MLP) and long short-term memory (LSTM) networks, to evaluate the impact of feature selection on predictive performance. The methodology is validated on a real-world photovoltaic (PV) dataset from southern Algeria, a region that has been largely overlooked in existing studies. Our results demonstrate that the proposed approach delivers strong predictive capabilities while remaining accessible and adaptable for practitioners and stakeholders in the energy sector.

The paper is organized as follows: in “Overview of the solar photovoltaic plant” section, describes the studied PV plant system. In “Methodology” section, presents the theoretical background of our proposed model. The experimental setup of our experiments is explained in “Experimental setup” section including the configuration of the proposed model, the data preprocessing and the model evaluation. Results and discussion are presented in “Results and discussion” section. Finally; “Conclusion” section offers the essential findings of this work, as well as some suggestions for future research.

## Overview of the solar photovoltaic plant

**Fig. 2:**
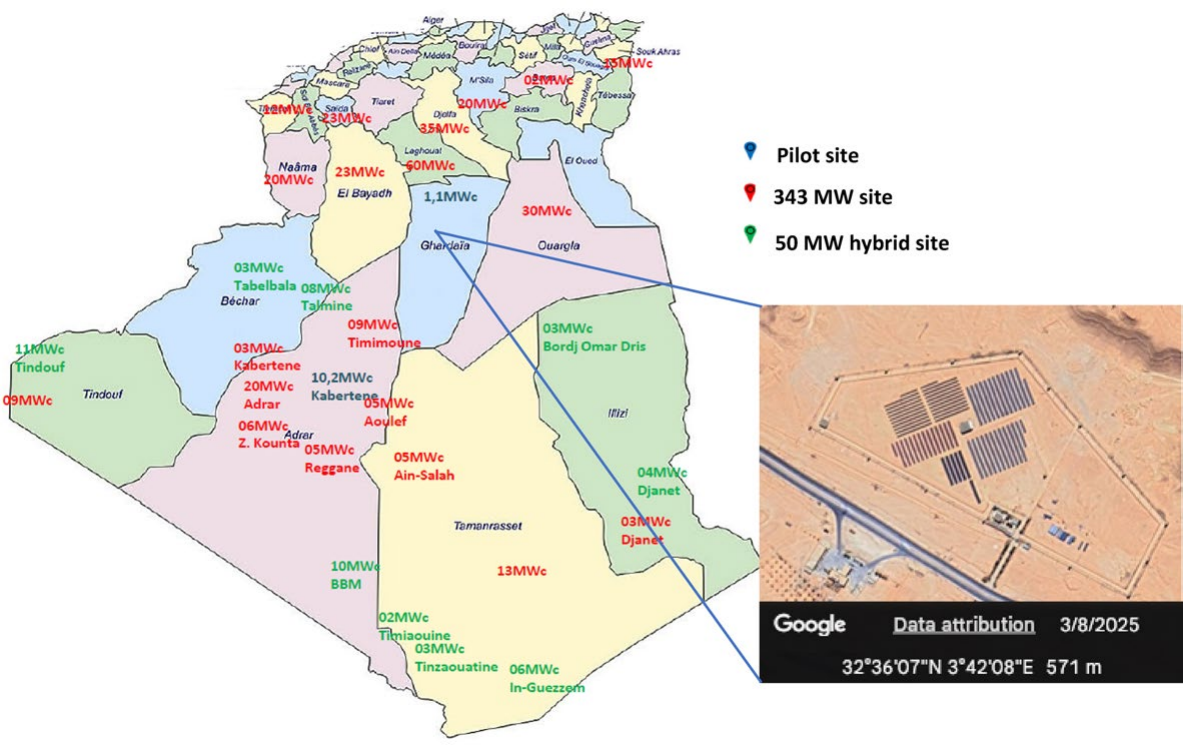
Studied site location of the PV central. Satellite imagery was obtained using Google Earth Pro (version 10.79.0.2, multi-threaded; Google LLC). Map imagery ©2025 Google, image ©2025 Maxar Technologies. Available at: https://earth.google.com/web/.

The study area focused on the PV power plants in the Ghardaia region of Algeria to validate the models. This region is home to a PV power plant operated by “SKTM”. Situated approximately 15 km north of the city of Ghardaia, near the village of OUED NECHOU, the plant features a semi-desert climate with geographic coordinates of 32° 29′ N 3° 40′ E and an altitude of 566 m. The geographical coordinates of the study site are depicted on the map of Algeria (see Fig. 2).

OUED NECHOU experiences solar irradiation reaching values of 900–1000 W/m^2^ during summer, typical of the Saharan climate characterized by high temperatures and sandstorms prevalent in southern regions. Serving as a pilot facility, this plant aims to assess the performance of solar equipment and its adaptation to the local climate. It has a nominal power capacity of approximately 1100 kWc.

Commissioned as part of Algeria’s National Renewable Energy Program in 2014, the solar photovoltaic plant in Ghardaia preceded the construction of 23 similar plants across the highlands and southern regions, collectively aiming to produce 400 megawatts. This Ghardaia plant utilizes multiple technologies, including thin amorphous silicon (a-Si) (Cd-Te), amorphous silicon (a-Si), polycrystalline silicon, and monocrystalline silicon (a-Si n la-Si).

Prior to model training and evaluation, a series of data preprocessing steps were performed to ensure the quality and reliability of the PV production dataset. Given the inherent challenges associated with real-world measurements, the raw data required careful cleaning and preparation as follows:

- *Outlier removal* Extreme or abnormal values inconsistent with typical PV power production behavior were identified and removed using threshold-based filtering methods to prevent skewing the model training process.
- *Handling missing values* Instances with missing values (NaNs) were addressed through linear interpolation, providing a smooth and continuous dataset without introducing bias.
- *Negative value elimination* Any negative PV power values, which are physically meaningless, were systematically excluded from the dataset.
- *Daylight data filtering* To focus the forecasting model on meaningful production periods, data was restricted to daylight hours only, specifically from sunrise to sunset, using solar position calculations to define the relevant time windows each day.

**Fig. 3:**
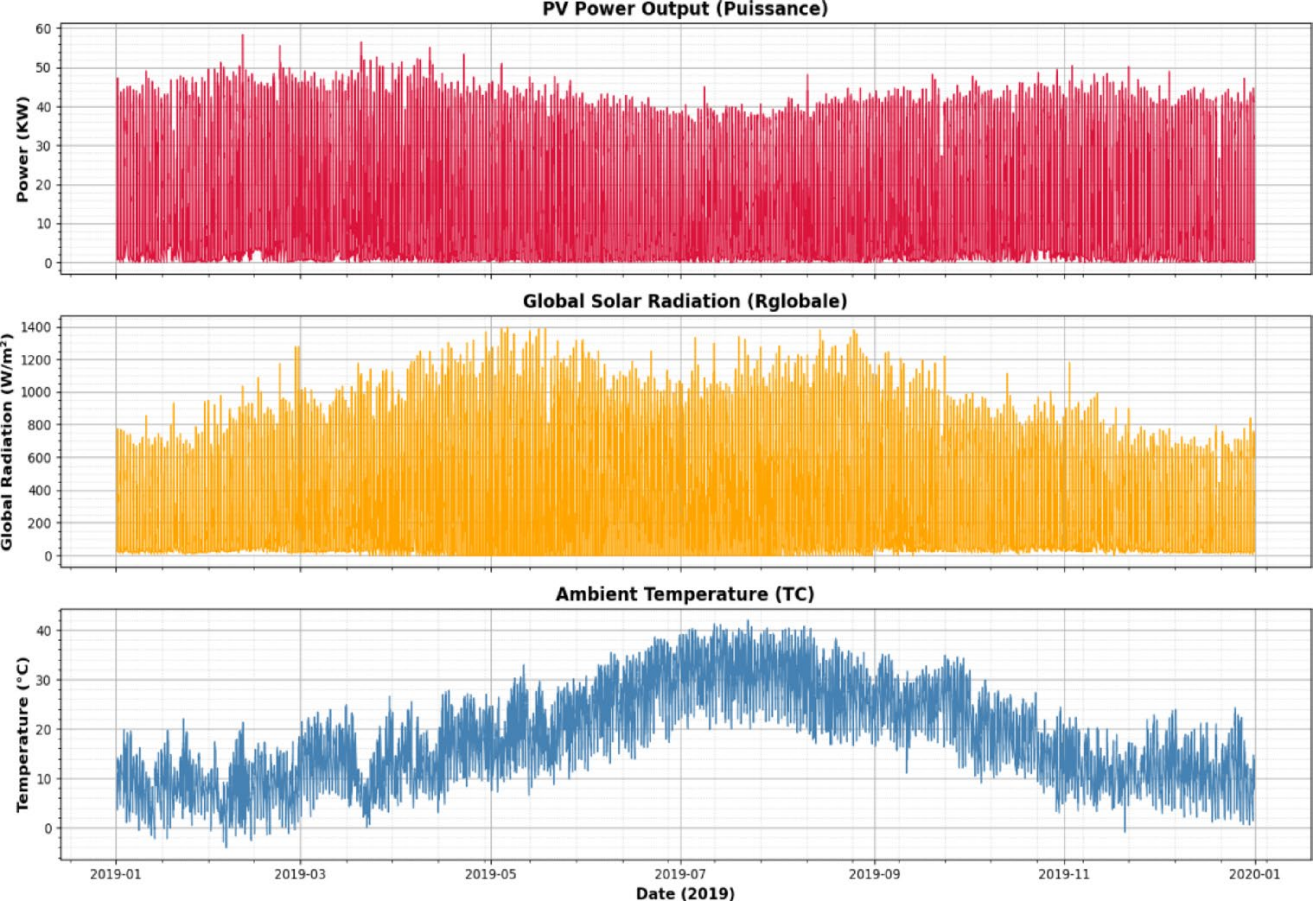
Time series data.

**Fig. 4:**
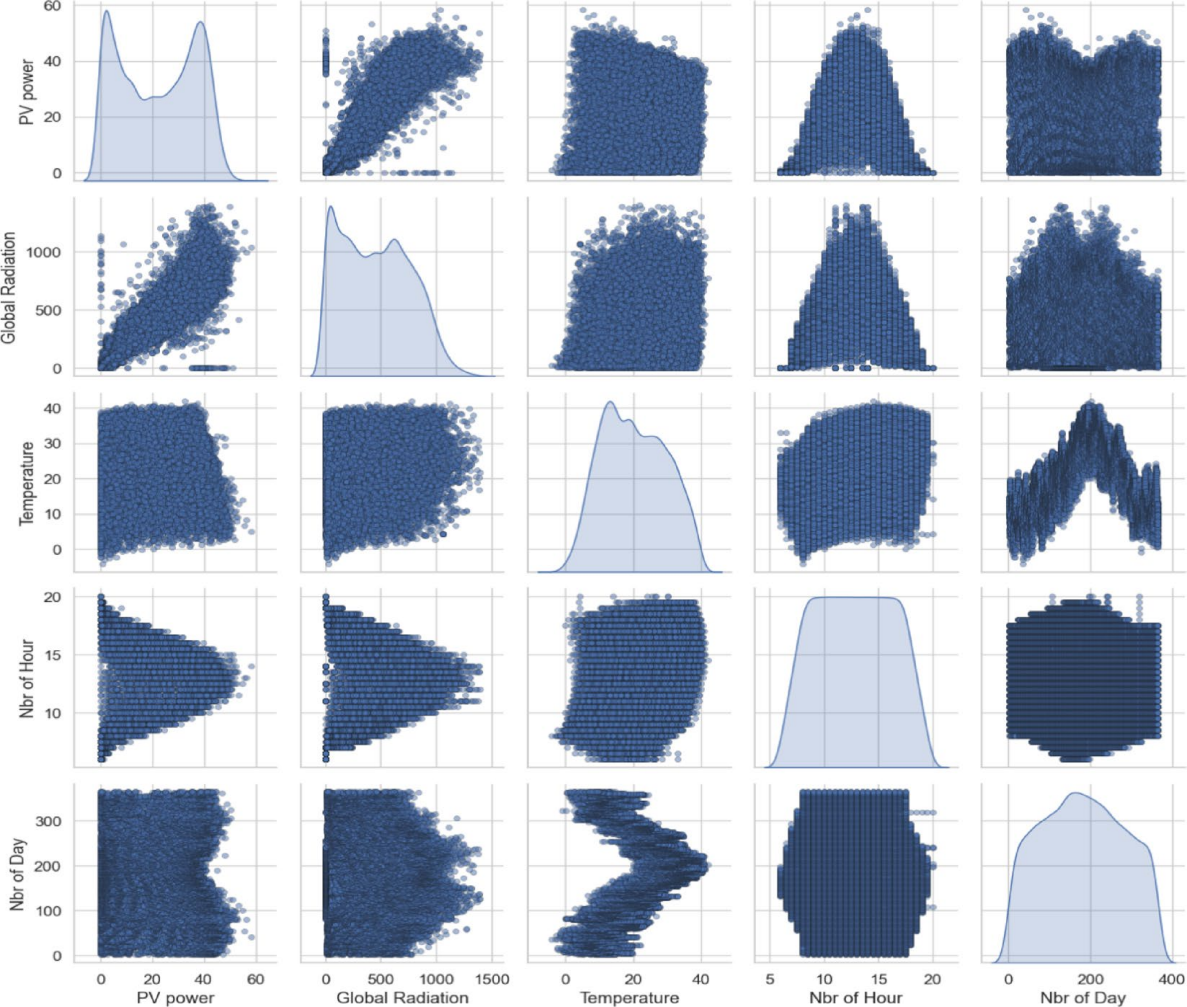
Pairwise correlation.

The time series plots illustrate the daily variations of PV power output, global solar radiation, and ambient temperature throughout 2019, highlighting seasonal patterns and the variability typical of intermittent solar data (Fig. 3). The pairwise correlation plots show the relationships and distributions among PV power, global radiation, temperature, hour of the day, and day of the year, indicating strong correlations between PV power and solar radiation (Fig. 4).

## Methodology

In this section, we will provide the theoretical background for various aspects addressed in this study. We delve into the intricate domain of feature selection techniques alongside our chosen forecasting tool, the MLP (multi-layer perceptron).

### Features selection

In typical scenarios, datasets employed for predicting PV power comprise multiple predictors, encompassing variables such as temperature (T), number of day (nD), number of hour (nH), and global solar radiation (GSR), among others. Given this plethora of features, feature selection techniques play a pivotal role in identifying the most pertinent predictors for constructing an effective prediction model. These techniques assist in ascertaining the relevance weight of each input parameter, commonly referred to as predictors, and their potential contribution to predicting the response variable.

The primary objective of our study, as previously articulated, is to identify the optimal combination of input parameters that yield accurate predictions of PV power. To accomplish this objective, we have employed various feature selection techniques. These techniques serve to streamline the input data by reducing dimensionality and eliminating redundant noisy data. By doing so, they effectively mitigate the complexity of the prediction process and enhance the performance of the predicted response.

#### ReliefF technique

The ReliefF algorithm, proposed by Kira and Rendell^19,20^, is a robust technique utilized in ML and data mining to identify relevant features for classification tasks. It systematically evaluates the discriminatory power of each feature by analyzing the differences in feature values between instances of the same and different classes.

During each iteration of the algorithm, the weight ($W_{i}$) assigned to each feature (*i*) is updated based on the observed differences. This update is governed by the following equation:

1 $$W_{i} = W_{i} - \left( {x_{i} - nearHIT_{i} } \right)^{2} + \left( {x_{i} - nearMISS_{i} } \right)^{2}$$

where $W_{i}$ represents the weight assigned to feature *i*. $x_{i}$ denotes the value of feature *i* in the current instance. $nearHIT_{i}$ and $nearMISS_{i}$ correspond to the values of feature *i* in the nearest neighbor instances belonging to the same and different classes, respectively.

The ReliefF algorithm iteratively computes these weights for all features in the dataset, assigning higher weights to features that exhibit larger differences between instances of the same and different classes.

One of the strengths of the ReliefF algorithm is its ability to handle datasets with mixed types of features, including both categorical and continuous variables. It achieves this by appropriately adjusting the computation of feature weights based on the data type. Furthermore, the ReliefF algorithm is known for its computational efficiency and scalability, making it well-suited for analyzing large datasets with high-dimensional feature spaces.

#### Minimum correlation technique

The minimum correlation features selection (minimum CFS) technique is a straightforward and efficient method used for feature selection in ML. It operates by analyzing the correlations between input features to identify the most relevant predictors for a given task. This technique is particularly useful when seeking to streamline datasets by selecting only the most informative features while minimizing redundancy and noise.

Assuming we have a dataset with n instances and p features, the minimum CFS computes a correlation matrix *C* of size *p* by *p*, where each element $C_{i,j}$ represents the correlation between features $f_{i}$ and $f_{j}$. Mathematically, this can be expressed as:

2 $$C = \left[ {\begin{array}{*{20}c} {C_{1,1} } & {\quad C_{1,2} } & {\quad \ldots } & {\quad C_{1,p} } \\ {C_{2,1} } & {\quad C_{2,2} } & {\quad \ldots } & {\quad C_{2,p} } \\ \vdots & {\quad \vdots } & {\quad \ddots } & {\quad \vdots } \\ {C_{p,1} } & {\quad C_{p,2} } & {\quad \ldots } & {\quad C_{p,p} } \\ \end{array} } \right].$$

To compute the weight $W_{i}$ of feature $f_{i}$ the minimum CFS technique calculates the mean correlation coefficients between feature $f_{i}$ and all other features. This is represented by the equation:

3 $$W_{i} = score_{i} \frac{1}{p}\sum\limits_{j = 1}^{p} {C_{i,j} }$$

The feature with the lowest mean correlation coefficient is considered the most relevant, as it is the least correlated with other features. Mathematically, this is determined by:

4 $$Best f_{i} = {\text{min}}\left( {{\text{mean}}\left( C \right)} \right)$$

Following this criterion, the mean correlation values are sorted in ascending order, and features with the lowest weights are selected as relevant predictors.

The minimum CFS technique is valued for its simplicity and effectiveness in identifying the most significant features while minimizing inter-feature correlations. It is a valuable tool for feature selection in various ML tasks, contributing to the construction of more efficient and accurate predictive models.

#### Chi-square test technique

The Chi-square test technique is a statistical method used for assessing the association between categorical variables^21^. It is particularly useful for analyzing contingency tables with two or more categories. The technique evaluates the independence between two variables by comparing observed frequencies to expected frequencies under the assumption of independence.

In the context of feature selection, the Chi-square test is applied to assess the relationship between individual features and the target variable. The test calculates a chi-squared statistic, denoted as *χ*^2^, which quantifies the discrepancy between observed and expected frequencies. A higher *χ*^2^ value indicates a stronger association between the feature and the target variable.

The formula for computing *χ*^2^ is:

5 $$\chi^{2} = \mathop \sum \limits_{i = 1}^{r} \mathop \sum \limits_{j = 1}^{c} \frac{{\left( {O_{ij} - E_{ij} } \right)^{2} }}{{E_{ij} }}$$

where $O_{ij}$ is the observed frequency and $E_{ij}$ is the expected frequency based on the null hypothesis. *r* and *c* represent the number of rows and columns in the contingency table, respectively.

After calculating *χ*^2^ for each feature, those with higher values are considered more relevant to the target variable and are typically selected for further analysis or model training. Conversely, features with lower *χ*^2^ values may be deemed less informative and can be excluded from the feature set.

#### The minimum redundancy maximum relevance technique

The minimum redundancy maximum relevance (mRMR) is a method for selecting features based on mutual information^22^. In information theory, mutual information measures the reduction in uncertainty about one random variable given knowledge of another.

For two random variables, *x* and *y*, mutual information is defined with respect to their probability density functions:

6 $$I\left( {x;y} \right) = \iint {p\left( {x,y} \right)\log \frac{{P\left( {x,y} \right)}}{p\left( x \right)p\left( y \right)}} dxdy$$

Using mutual information, feature relevance and redundancy are defined as follows:

7 $$Redundancy\;\left( S \right) = \frac{1}{{\left| S \right|^{2} }}\mathop \sum \limits_{{f_{i,} f_{j} \in S}} I\left( {f_{i} ,f_{j} } \right)$$

8 $$Relevance\;\left( {S,c} \right) = \frac{1}{\left| S \right|}\mathop \sum \limits_{{f_{i} \in S}} I\left( {f_{i} ,c} \right)$$

where *S* is the set of features, *c* is the sample class, and *f* represents individual features in *S*.

The mRMR ranks features by simultaneously minimizing redundancy and maximizing relevance using an operator *Φ*:

9 $$\max \Phi \left( {Relevance,Redundancy} \right) = Relevance - Redundancy$$

mRMR feature selection is a powerful technique for identifying informative features in high-dimensional data sets while minimizing redundancy. It strikes a balance between relevance and redundancy, leading to improved model performance and interpretability in various ML tasks.

#### F-test technique

F-test is a statistical procedure that computes an f-score by comparing variances. In this study, we employ the f-test within a one-way analysis of variance (ANOVA) framework. ANOVA assesses the ratio of variance among different groups to the variance within each group, where groups are defined by instances sharing the same target value. A higher f-score suggests that the differences within groups are minimal compared to the differences between groups^23^. In this ANOVA-based feature selection approach utilizing the f-test, features are prioritized based on higher *f-score* values. The f-score in this context is expressed as:

10 $$f{\text{-}}score = \frac{Variance \;between\;groups}{{Variance\; within\; groups}}$$

where variance between groups represents the variability among groups defined by different feature values. Variance within groups represents the cumulative variability within individual groups. A higher F-score suggests that the feature is more relevant for distinguishing between groups, making it a valuable tool in feature selection for identifying significant predictors in a dataset.

#### Neighborhood component analysis technique

Neighborhood component analysis (NCA) is a feature selection technique that aims to optimize the prediction accuracy of regression algorithms by learning a distance metric. Developed by Jacob Goldberger et al.^24^, NCA transforms input data to maximize average leave-one-out (LOO) classification performance. It addresses drawbacks of the K-nearest neighbors (KNN) algorithm by utilizing a quadratic distance metric, reducing dimensionality, and improving computational efficiency. NCA’s features selection approach is particularly effective for regression tasks, where it enhances prediction accuracy by selecting relevant features.

#### Laplacian technique

Laplacian feature selection (LapFS) is a method employed in feature selection, particularly suited for high-dimensional datasets, aiming to pinpoint the most pertinent features relevant to a specific task.

At its core, LapFS relies on the Laplacian score, a metric designed to gauge the local structure of data points within the feature space. This score quantifies the similarity between data points based on their respective feature values, with higher Laplacian scores indicating greater importance or informativeness of features.

Widely applicable across diverse domains and tasks, LapFS stands as a robust technique for distilling meaningful insights from complex datasets. By harnessing the local structure of data points, it facilitates a targeted and efficient feature selection process, ultimately enhancing the performance of ML models.

In this work, feature selection techniques were chosen for their statistical efficiency and low computational cost. ReliefF ranks features based on nearest-neighbor distances; Minimum Correlation selects features with the least redundancy; Chi-square identifies features most associated with the target; mRMR balances maximum relevance and minimum redundancy using mutual information; F-test prioritizes features with the highest variance between groups; NCA learns feature weights to maximize predictive accuracy; and Laplacian Score preserves local data structures. For each method, features were ranked and models were trained incrementally (using 1–4 features), with the optimal subset selected based on achieving the highest R^2^ and the lowest normalized MAE (nMAE).

### Multi-layer perceptron neural network

A multilayer perceptron (MLP) is a type of ANN that consists of multiple layers of perceptrons. These layers typically include an input layer, one or more hidden layers, and an output layer. Each perceptron in the network is connected to every perceptron in the adjacent layers, and each connection has an associated weight.

**Fig. 5:**
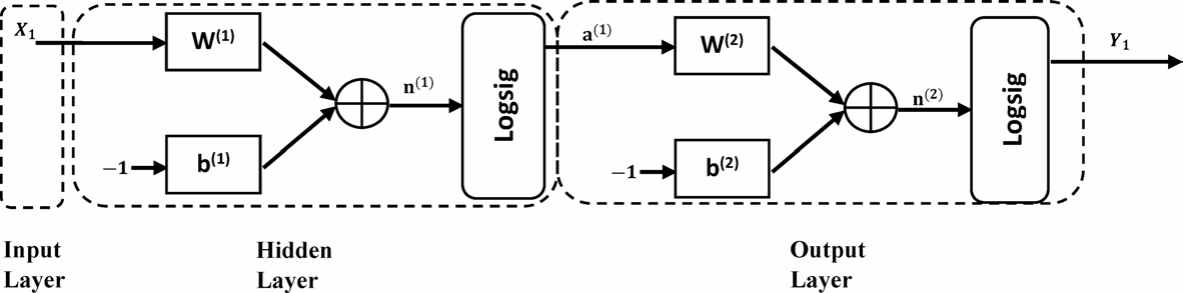
Feed-forward neural network MLP model.

The architecture of a generic MLP model, as illustrated in Fig. 5, is commonly used for supervised learning tasks. In supervised learning, the MLP learns to model the relationship between input data and corresponding output labels or predictions. During training, the model adjusts its weights and biases to minimize the error between its predictions and the true output labels (Eq. 11).

Training an MLP involves using backpropagation algorithms, such as gradient descent, conjugate gradient, or Levenberg–Marquardt (LM), to update the weights and biases based on the error calculated during each iteration of training. This process iteratively adjusts the parameters of the network to improve its performance on the training data.

11 $$y_{j} = f\left( {\left( {\mathop \sum \limits_{i} w_{ij} *X_{ij} } \right) - b_{j} } \right)$$

where $y_{j}$ is the output, *X* is the input data, $w_{ij}$ is the weights vector, and $b_{j}$ is the bias.

### Long short-term memory (LSTM) networks

**Fig. 6:**
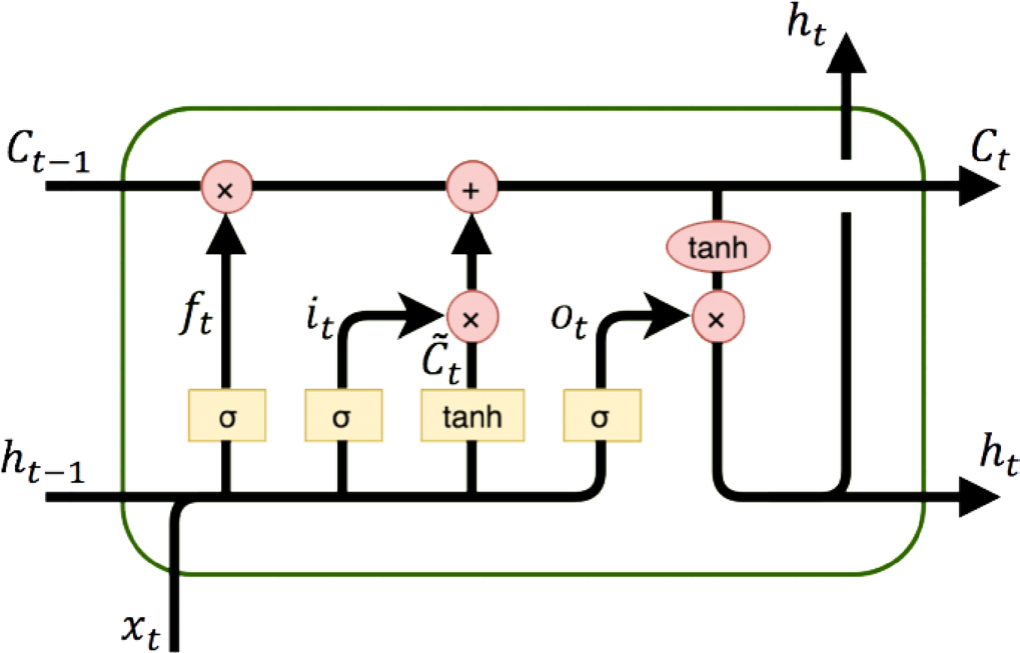
LSTM model.

Long short-term memory (LSTM) networks, as illustrated in Fig. 6, a specialized form of recurrent neural networks (RNNs), have demonstrated remarkable performance in forecasting time series data characterized by non-linearity and temporal dependencies. Originally proposed by Hochreiter and Schmidhuber in 1997, LSTM networks are specifically designed to overcome the limitations of traditional RNNs, such as vanishing and exploding gradients, by incorporating memory cells and gating mechanisms that regulate the flow of information.

In the context of renewable energy forecasting, particularly for intermittent and highly variable resources such as photovoltaic (PV) power, wind speed, and solar radiation, LSTM networks have proven to be highly effective. Their ability to learn long-term temporal patterns enables them to capture the underlying dynamics of fluctuating meteorological and production data more accurately than conventional feed-forward networks.

Recent studies have consistently highlighted the superiority of LSTM-based models in predicting intermittent energy sources. For instance, LSTM models have been successfully employed to forecast PV power output by modeling sequential patterns in solar irradiance and temperature data. Similarly, in wind energy forecasting, LSTM networks have demonstrated enhanced predictive accuracy by learning the stochastic behavior of wind speed over time. In the case of solar radiation forecasting, LSTM models efficiently capture diurnal and seasonal variations, leading to improved reliability compared to traditional machine learning methods.

The key strengths of LSTM networks in renewable energy forecasting include:

- *Temporal memory* LSTMs retain historical information across long time horizons, crucial for accurately predicting slow-evolving weather patterns.
- *Nonlinear mapping* They effectively model complex, nonlinear relationships inherent in renewable energy datasets.
- *Robustness to noise* LSTM networks are capable of filtering out noise in highly variable data such as PV output under cloudy conditions or wind speed fluctuations.
- *Adaptability* They perform well across different climatic regions and operational scenarios, making them suitable for diverse forecasting applications.

In this study, an LSTM model is integrated alongside the traditional ANN approach to provide a benchmark comparison, highlighting the advantage of sequence modeling for PV power forecasting tasks. The inclusion of LSTM aims to enhance the robustness and generalization of the forecasting system, particularly under conditions of high variability and uncertainty.

## Experimental setup

In this section, we will offer insights into the dataset utilized for our analysis, presenting a comprehensive overview, aside with the used data normalization process. In addition, the opted configuration of the selected forecasting tool in this work, which is the MLP, is explained. Finally, we discuss the evaluation metrics meticulously selected to assess the robustness and efficacy of our research outcomes.

### Data pre processing

As outlined in the previous section, the dataset comprises multiple input features, including temperature and global irradiation, and a single output representing PV power. In this study, each feature undergoes linear scaling to normalize its values within the range[0, 1], following the equation:

$$Xnor_{i} = \left( {X_{i} - \min \left( X \right)} \right)/\left( {\max \left( X \right) - \min \left( X \right)} \right)$$

Here, *X* denotes the input data vector, $Xnor_{i}$ represents the normalized value of input $X_{i}$, and min/max correspond to the minimum and maximum values within *X*.

Similarly, the output data (PV power) is scaled to fit within the range of 0–1. Subsequently, the dataset is divided into three subsets: 70% for training, 15% for validation, and the remaining portion for testing. The training set is utilized to fine-tune the model hyperparameters, the test set is employed to evaluate the model’s performance, and the validation set serves to prevent overfitting.

**Fig. 7:**
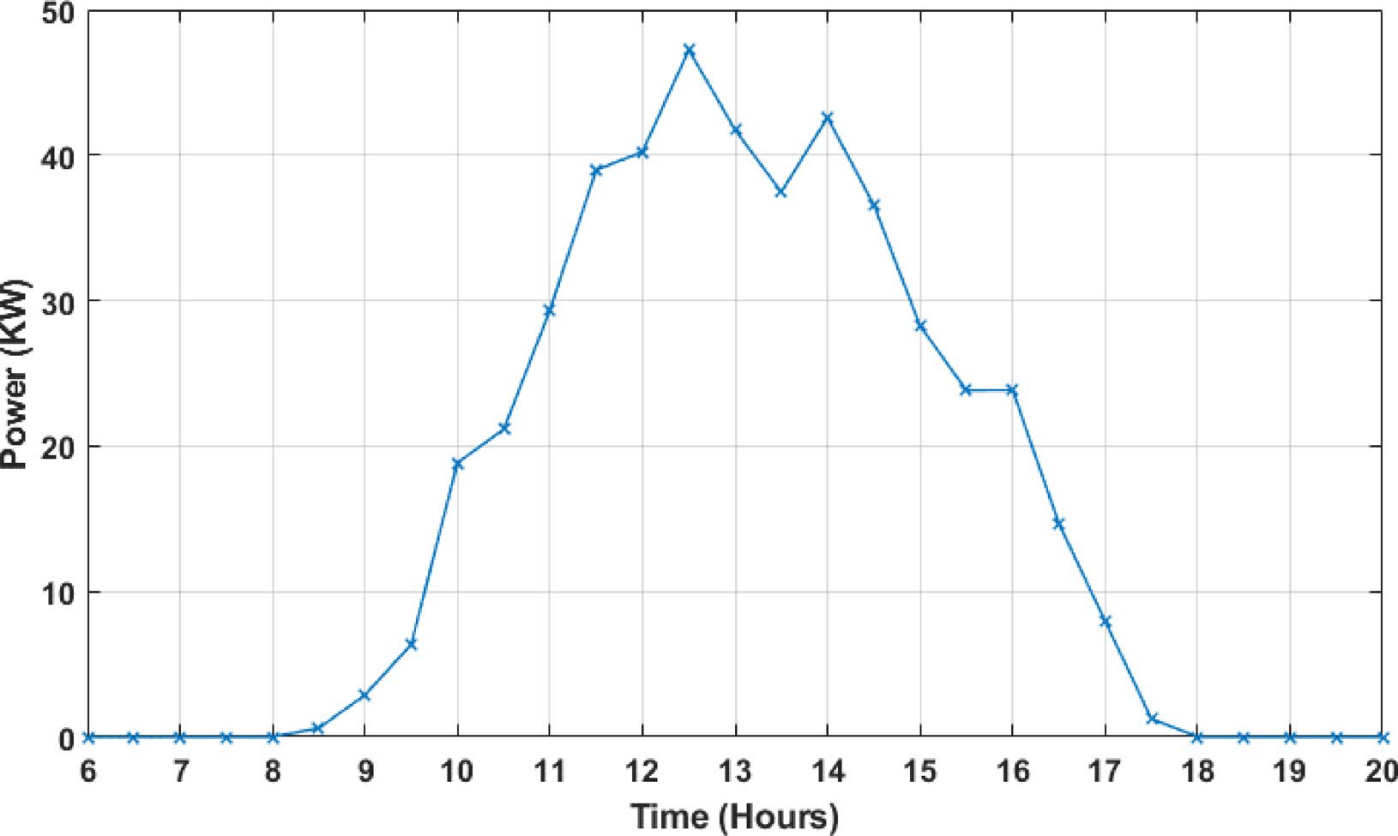
PV power variation in function of the time for a period of 1 day (01/01/2018).

**Fig. 8:**
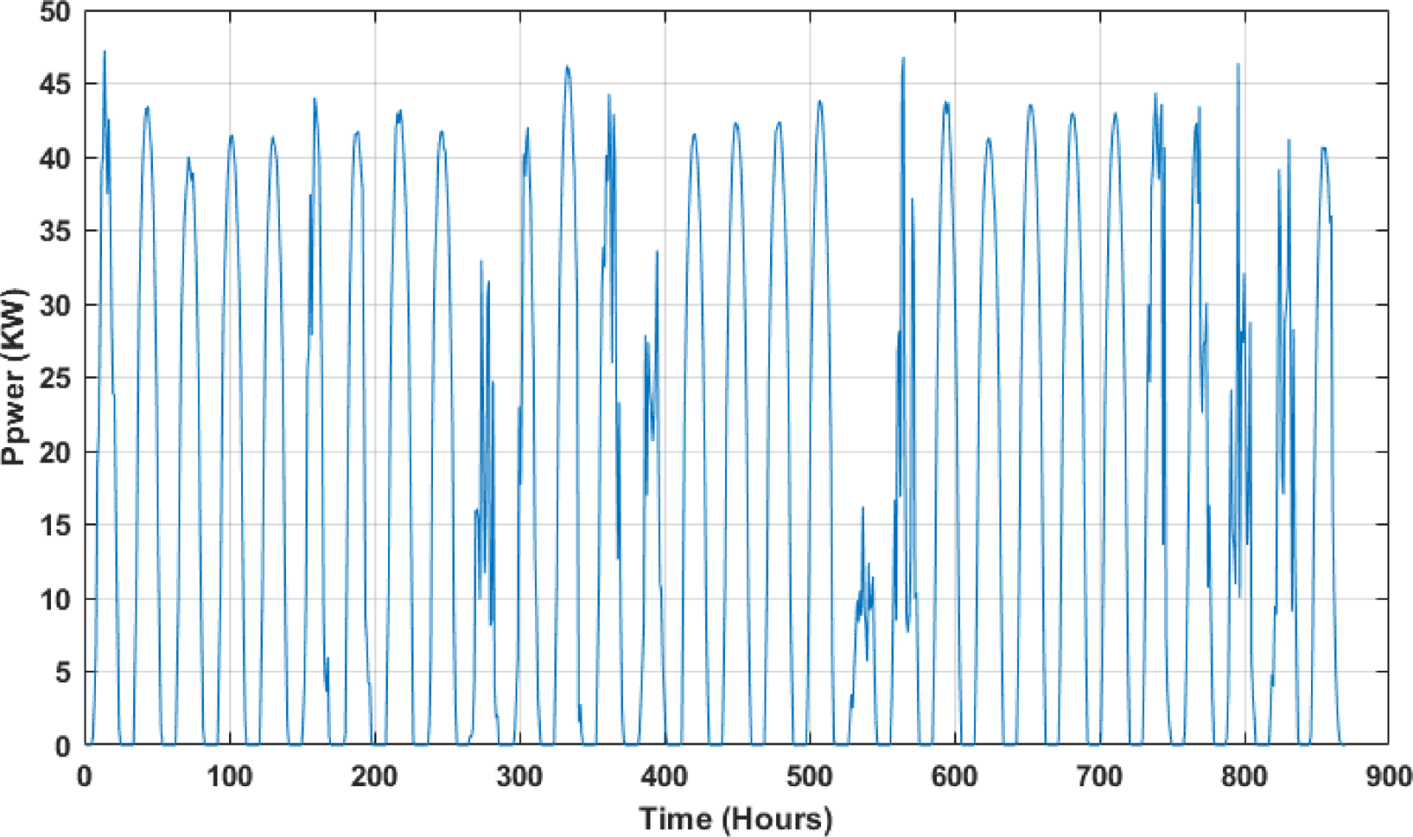
PV power variation in function of the time for a period of 1 Month (January 2018).

Figures 7 and 8 present samples of the variation of the PV power in function of the time for periods of 1 day (01/01/2018) and 1 month (January 2018), respectively.

### Models’ setup

In this study, we utilized both a multilayer perceptron (MLP) and a long short-term memory (LSTM) network for forecasting PV power. We selected MLPs due to their robust predictive capabilities in learning highly non-linear relationships between input and output data. The specific MLP architecture employed consists of two hidden layers, each containing 10 neurons with sigmoid activation functions, followed by an output layer with a single linear neuron. This configuration was determined through iterative experimentation, testing various numbers of layers and neurons per layer. To train the MLP, we employed the Levenberg–Marquardt backpropagation algorithm, chosen for its efficiency and fast convergence, particularly beneficial for limited training data. Alongside the MLP, we incorporated an LSTM network to better capture the temporal dependencies inherent in PV power generation. The LSTM model architecture consists of two stacked LSTM layers with 64 and 32 units respectively, both using tanh activations, followed by a linear dense output layer. This stacked design offered an optimal balance between complexity and generalization. For training the LSTM, input sequences were normalized using a Min–Max scaler, and the Adam optimizer with an adaptive learning rate was applied. Final evaluations for both models were performed after inverse-scaling the outputs to retain interpretability in the original physical units (kW).

### Model validation

Evaluating the accuracy of machine learning models is crucial for understanding their ability to make reliable predictions. This evaluation involves comparing the predicted values with the actual measured data using several statistical performance metrics. In this study, we employed four key statistical indices to assess the performance of the proposed models: mean absolute error (MAE), normalized mean absolute error (nMAE), the coefficient of determination (R^2^), and the Pearson correlation coefficient (R). The equations and detailed descriptions of these metrics are summarized in Table 1.

**Table 1:** Statistical metrics used in this work.

Metrics	Equation	Description
MAE	$\sqrt{\frac{1}{n}\sum\limits_{i=1}^{n}\left|{H}_{E}-{H}_{M}\right|}$	Represents the sample standard deviation of the differences between the predicted values and the observed values. RMSE penalizes larger differences more than the average absolute difference. A lower RMSE indicates better model performance^25–29^
nMAE %	$\frac{MAE}{\frac{1}{N}\sum_{i=1}^{n}{H}_{M}}\times 100$	Provides a normalized measure of MAE relative to the mean of the observed values, expressed as a percentage. It allows easy comparison across different datasets or models. The model performance is categorized as follows^30–35^: Excellent if nMAE < 10%, Good if 10% ≤ nMAE < 20%, Fair if 20% ≤ nMAE < 30%, and Poor if nMAE ≥ 30%
$R$	$\frac{\sum_{i=1}^{n}\left({H}_{E}-{\overline{H} }_{E}\right).\left({H}_{M}-{\overline{H} }_{M}\right)}{\sqrt{\sum_{i=1}^{n}\left({H}_{E}-{\overline{H} }_{E}\right).\sum_{i=1}^{n}\left({H}_{M}-{\overline{H} }_{M}\right).}}$	Represents the coefficient of how well the predicted values fit compared to the original values. The value ranges from 0 to 1, where higher values indicate better model performance. R is a relative metric, allowing comparison between models trained on the same data^36–41^

These metrics, along with their equations and descriptions, are provided in Table 1. Here, $H_{E}$ represents the estimated value, $H_{M}$ denotes the measured values, and $\overline{H }$ denotes the mean value.

## Results and discussion

In this study, we systematically assessed the impact of feature selection techniques on the forecasting performance of two machine learning models: the multilayer perceptron (MLP) and the long short-term memory (LSTM) network. The evaluation was based on several statistical metrics, namely mean absolute error (MAE), normalized mean absolute error (nMAE), coefficient of determination (R^2^), and Pearson correlation coefficient (R).

Tables 3 and 4 summarize the results obtained for both models, respectively.

### Baseline performance (all inputs, without feature selection)

- *MLP (All Inputs)* achieved an MAE of 2.194 kW, an nMAE of 9.965%, an R^2^ of 0.942, and an R of 0.970.
- *LSTM (All Inputs)* slightly outperformed MLP with an MAE of 2.108 kW, an nMAE of 9.573%, an R^2^ of 0.944, and an R of 0.972.

This indicates that when using *all input features*, the LSTM model provides *marginally better predictive performance* compared to MLP, likely owing to LSTM’s superior ability to model complex temporal dependencies and dynamic patterns, even in non-time-sequential datasets.

### Impact of feature selection techniques

When applying feature selection (using only top-ranked predictors from each method), we observed several important trends:

- *Overall improvement* In many cases, especially when selecting 3 or 4 features, feature selection improved or maintained model performance compared to using all features.
- *Best feature selection methods* Across both MLP and LSTM models, techniques such as *ReliefF*, *MRMR*, *NCA*, and *Mutual Information (mRMR)* led to the highest performance improvements.
- *Number of features* As expected, performance generally improved when moving from 1 to 3 or 4 selected features. Using only 1 feature typically caused a *notable drop* in performance, but including more relevant features (up to 4) restored and sometimes enhanced the prediction quality.
- With *ReliefF* (4 features), MLP achieved an MAE of 1.6018 kW, an nMAE of 9.2143%, and an R^2^ of 0.9608—significantly better than the baseline using all inputs.
- *MRMR*, *Chi-square*, and *NCA* also yielded excellent results at 4 features, pushing R^2^ values consistently above 0.960.
- Feature selection helped *reduce MAE* by approximately 25–30% compared to using all features.
- *Minimal correlation* and *Chi-square* feature selections performed less effectively when only 1 or 2 features were selected but improved substantially at 3 or 4 features.
- LSTM benefited similarly from feature selection, but the improvements were slightly less dramatic compared to MLP because the baseline LSTM performance was already strong.
- Using *ReliefF* with 4 features, LSTM achieved an MAE of 2.144 kW, an nMAE of 9.735%, and an R^2^ of 0.945—nearly identical to the baseline.
- Interestingly, *Mutual Information (mRMR)* and *NCA* allowed the LSTM model to maintain an R^2^ of approximately 0.945–0.946 while slightly lowering MAE compared to the no-feature-selection baseline.
- Selecting only 1 feature caused a *major drop* in performance across all techniques, especially for Chi-square and Minimum Correlation, where R^2^ fell dramatically below 0.70 in some cases.
- *MLP* is more *sensitive to feature selection*; careful reduction to the most informative features significantly boosts its forecasting accuracy.
- *LSTM* exhibits *greater robustness* to feature selection: even when fewer features are used, it maintains relatively strong performance.
- The *best overall performance* for MLP was achieved with *ReliefF* and *MRMR* using 4 features.
- For LSTM, *minimal gains* were observed from feature selection, suggesting that LSTM can inherently manage redundant or less informative inputs better than MLP.

Table 2 presents the effectiveness of daily photovoltaic power forecasting when different predictors are selected using various feature selection techniques and MLP model, highlighting the relationship between predictor choices and forecasting accuracy.

**Table 2:** The performance of daily PV power forecasting as a function of the number of selected predictors using different feature selection techniques Integrated with MLP model.

	Num_Features	MAE	nMAE	R_square	R
MLP (All inputs)	4	2.194	9.965	0.942	0.97
MLP-Releiff	1	4.0850	23.4989	0.8477	0.9207
	2	2.3455	13.4927	0.9404	0.9697
	3	1.7237	9.9154	0.9591	0.9793
	4	**1.6018**	**9.2143**	**0.9608**	**0.9802**
MLP-Min correlation	1	4.3737	25.1601	0.7995	0.8941
	2	3.9726	22.8525	0.8190	0.9050
	3	1.7568	10.1059	**0.9593**	**0.9795**
	4	**1.7275**	**9.9375**	0.9593	0.9795
MLP-Chi-squared	1	4.3814	25.2040	0.7994	0.8941
	2	2.3496	13.5161	0.9403	0.9697
	3	1.7211	9.9007	**0.9587**	0.9791
	4	**1.6254**	**9.3501**	0.9605	**0.9801**
MLP-MRMR	1	4.0992	23.5810	0.8481	0.9209
	2	3.0914	17.7836	0.9086	0.9532
	3	1.7061	9.8145	**0.9599**	**0.9797**
	4	**1.6609**	**9.5545**	0.9600	0.9798
MLP-F-test	1	4.3860	25.2304	0.7994	0.8941
	2	2.3288	13.3966	0.9406	0.9698
	3	1.7064	9.8162	**0.9594**	0.9795
	4	**1.6523**	**9.5050**	0.9604	**0.9800**
MLP-NCA	1	4.0945	23.5536	0.8479	0.9208
	2	2.3351	13.4328	0.9410	0.9701
	3	1.7141	9.8601	0.9596	0.9796
	4	**1.6509**	**9.4968**	**0.9610**	**0.9803**
MLP-Laplacian	1	4.3809	25.2013	0.7994	0.8941
	2	2.3356	13.4355	0.9409	0.9700
	3	1.7360	9.9861	0.9591	0.9793
	4	**1.6699**	**9.6061**	**0.9607**	**0.9801**

Significant values are in [bold].

Table 3 demonstrates the effectiveness of daily photovoltaic power forecasting when different predictors are selected using various feature selection techniques and LSTM model, highlighting the relationship between predictor choice and forecasting accuracy.

**Table 3:** The performance of daily PV power forecasting as a function of the number of selected predictors using different feature selection techniques integrated with LSTM model.

Method	Num_Features	MAE_kW	nMAE_%	R2	R
LSTM (All inputs)	4	2.108	9.573	0.944	0.972
LSTM-Chi-square	1	13.166	59.795	0.011	0.107
	2	5.725	25.999	0.701	0.837
	3	5.434	24.68	0.723	0.85
	**4**	**2.046**	**9.29**	**0.946**	**0.973**
LSTM-F-test	1	4.633	21.04	0.811	0.901
	2	3.745	17.008	0.864	0.929
	3	3.039	13.801	0.897	0.947
	4	2.174	9.875	0.943	0.971
LSTM-Laplacian	1	4.595	20.87	0.811	0.901
	2	3.758	17.066	0.865	0.93
	3	3.082	13.996	0.897	0.947
	4	2.185	9.921	0.943	0.971
LSTM-Lasso (ReliefF ap.)	1	4.645	21.097	0.813	0.901
	2	3.727	16.927	0.863	0.929
	3	2.418	10.98	0.935	0.967
	4	2.104	9.555	0.945	0.972
LSTM-Minimum corr (inv.)	1	6.192	28.12	0.681	0.825
	2	5.795	26.32	0.701	0.837
	3	2.294	10.418	0.94	0.97
	4	2.143	9.733	0.944	0.971
LSTM-Mutual info (mRMR)	1	4.614	20.957	0.814	0.902
	2	2.905	13.192	0.918	0.958
	3	2.168	9.846	0.944	0.972
	4	2.114	9.602	0.945	0.972
LSTM-NCA	1	4.575	20.779	0.811	0.901
	2	3.729	16.938	0.865	0.93
	3	3.036	13.786	0.899	0.948
	4	2.089	9.489	0.944	0.972
LSTM-ReliefF	1	4.627	21.016	0.812	0.901
	2	3.759	17.072	0.865	0.93
	3	2.399	10.895	0.936	0.967
	4	2.144	9.735	0.945	0.972

Significant values are in [bold].

**Fig. 9:**
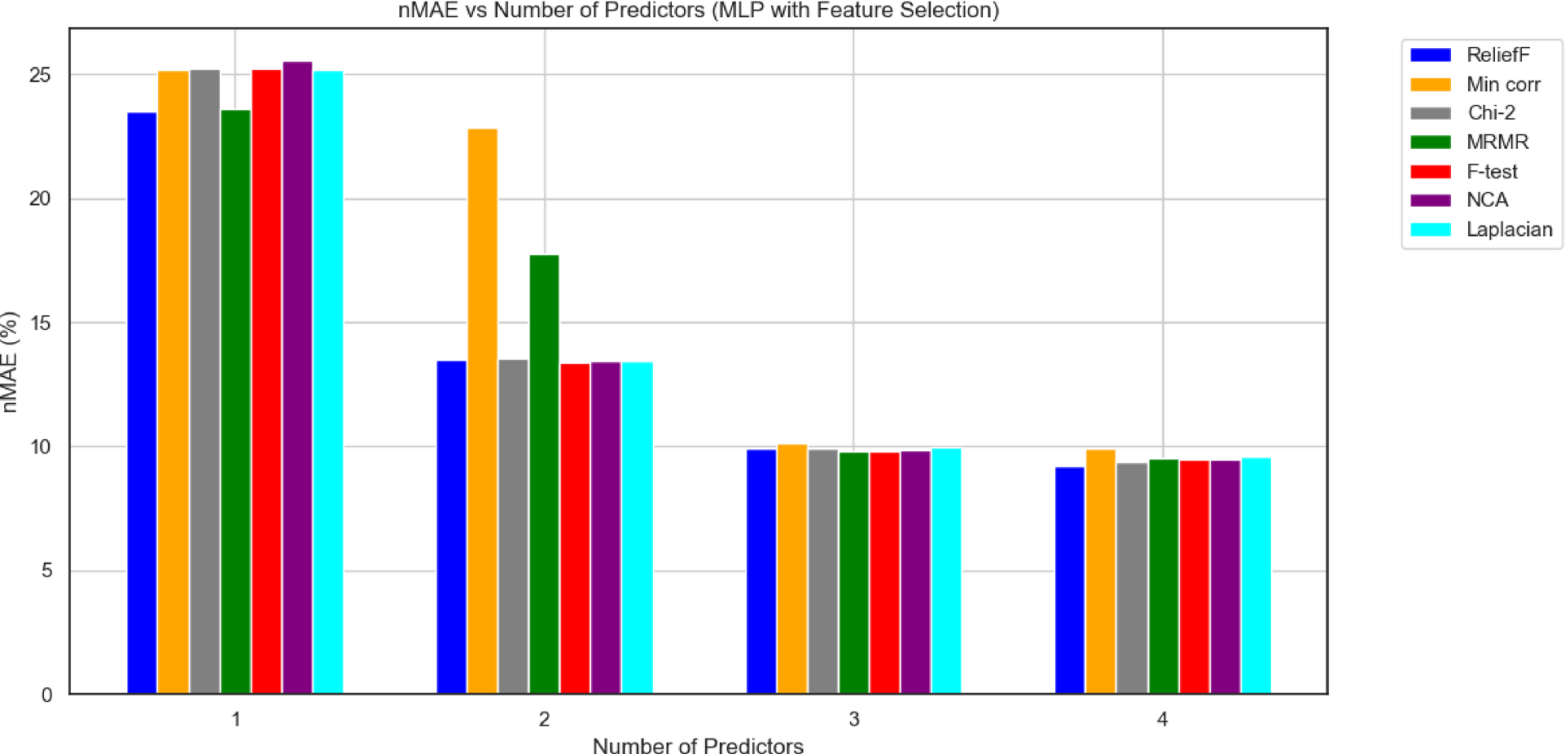
The nMAE in function of the number of selected predictors for the seven different used features selection techniques MLP_model.

**Fig. 10:**
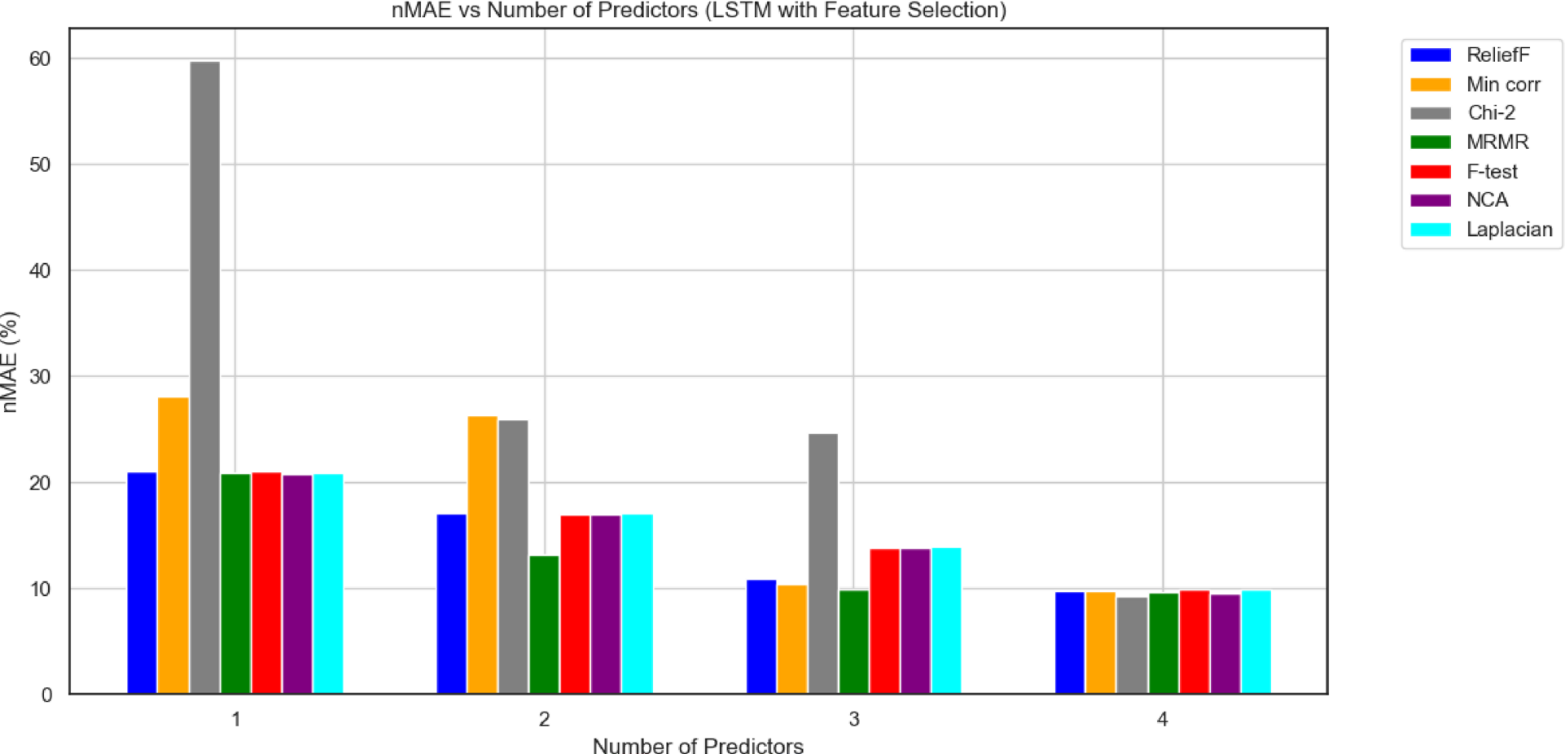
The nMAE in function of the number of selected predictors for the seven different used features selection techniques LSTM_model.

Graphical results for both models are presented in Figs. 9 and 10, respectively, in terms of the nMAE metric.

**Fig. 11:**
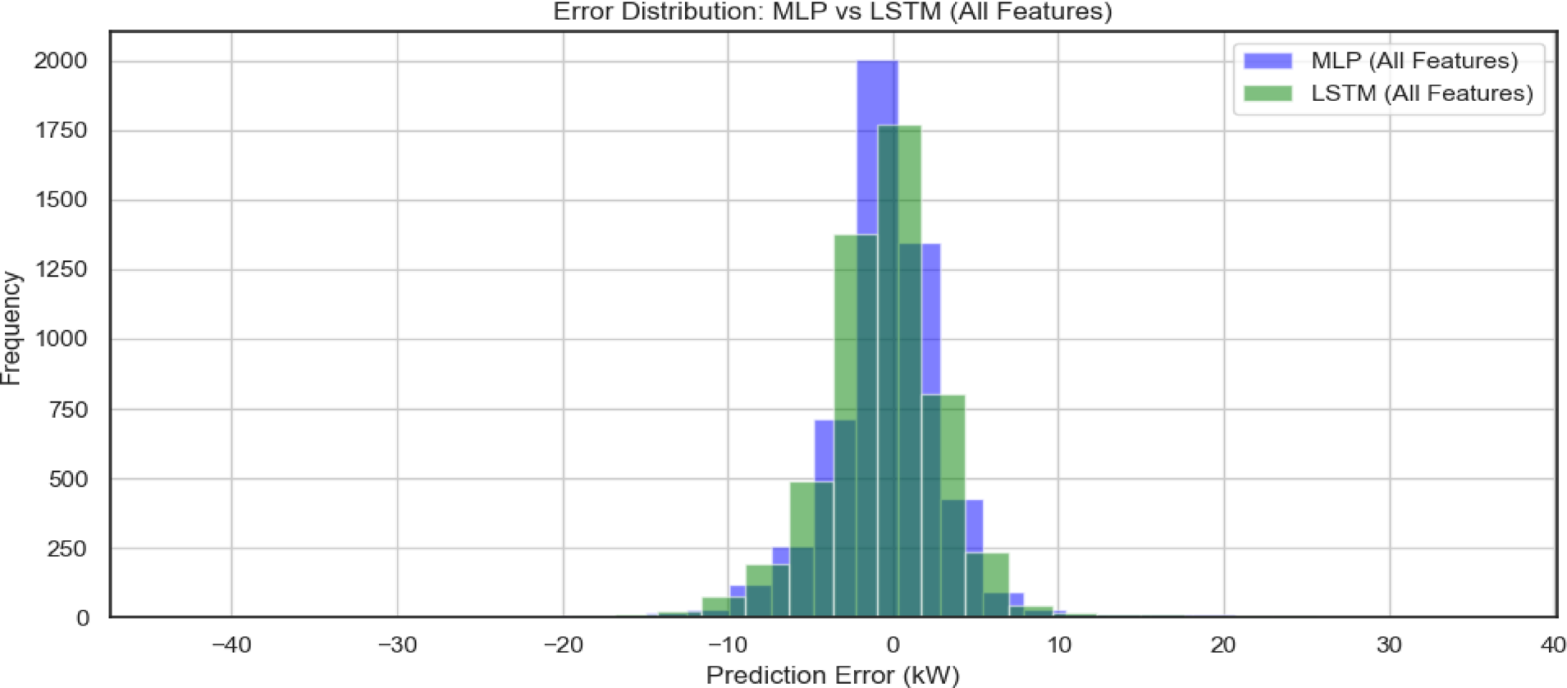
Error Distribution MLP against LSTM.

Figure 11, illustrates the error distribution of the MLP and LSTM models when utilizing *all available input features* for PV power forecasting. Both models exhibit a strong concentration of prediction errors around zero, indicating overall good predictive performance. However, the LSTM model demonstrates a slightly narrower and more peaked distribution, suggesting fewer extreme errors and a slightly better error consistency compared to the MLP.

**Fig.12:**
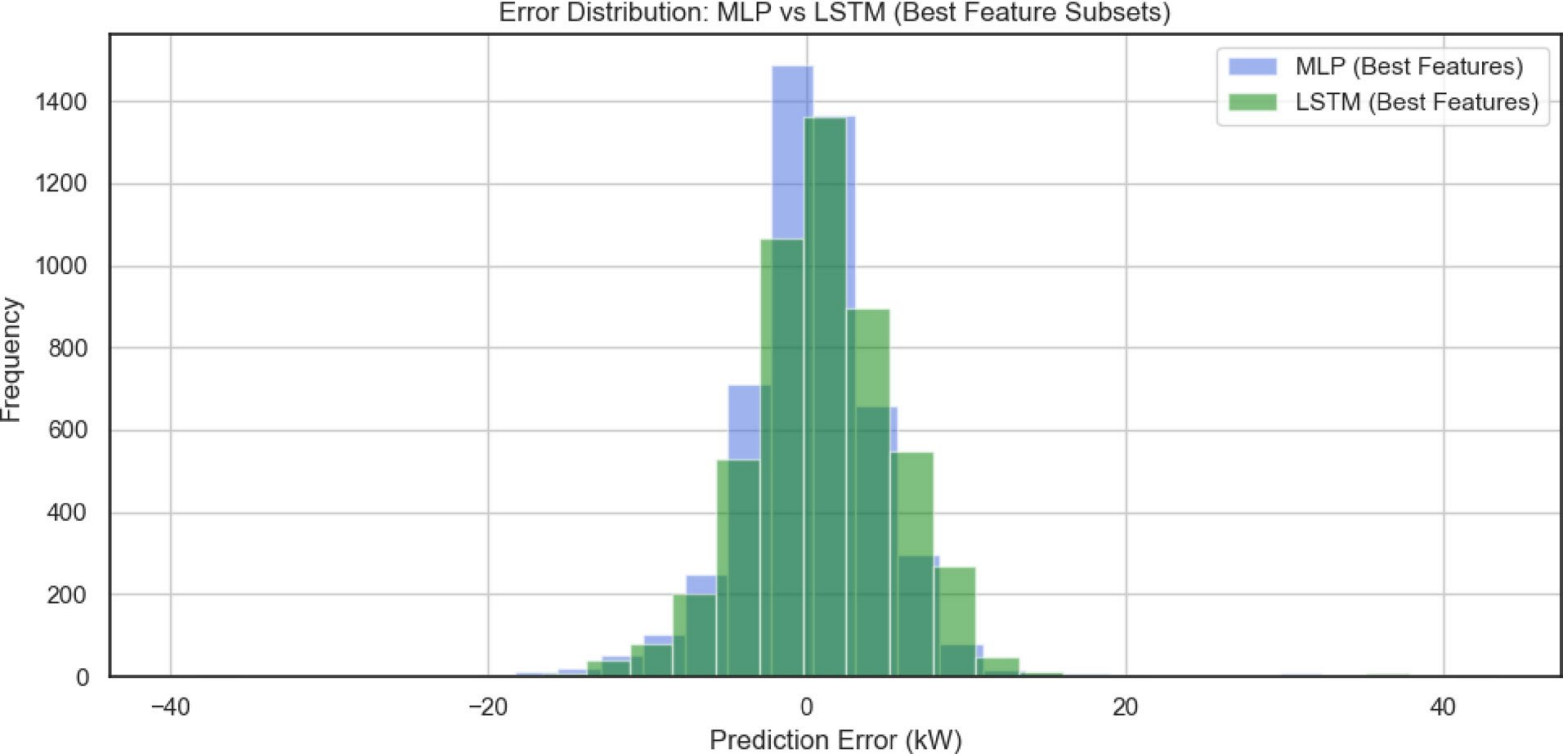
Error distribution MLP against LSTM bast feature case.

Figure 12, compares the error distributions of MLP and LSTM models when trained using *the best-ranked feature subsets* identified through feature selection techniques. Here, both models show a further tightening of the error distribution around zero relative to the all-feature case, highlighting the positive impact of feature selection. Notably, the LSTM model maintains a sharper peak with fewer large errors, confirming its superior generalization ability compared to the MLP under optimized input configurations.

The comparison between the best-performing models (Table4), MLP integrated with ReliefF feature selection (ReleifF_MLP), Random Forest, and LSTM integrated with Chi-square feature selection (Chi-square-LSTM), highlights clear trends in forecasting daily PV power production.

**Table 4:** Benchmarking the best models for daily PV output forecasting.

Model	MAE	nMAE	R^2^	R
ReleifF_MLP	**1.6018**	**9.2143**	**0.9608**	**0.9802**
Random forest	1.8271	9.2977	0.9528	0.9761
Chi-square-LSTM	2.046	9.29	0.946	0.973

Significant values are in [bold].

The ReleifF_MLP model achieved the lowest MAE (1.6018 kW) and highest R^2^ (0.9608) and correlation (R = 0.9802), outperforming both Random Forest and Chi-square-LSTM models. Random Forest followed closely with an MAE of 1.8271 kW and R^2^ of 0.9528, demonstrating robust predictive capabilities as a traditional ensemble learning method.

Chi-square-LSTM, although performing slightly worse with an MAE of 2.046 kW and R^2^ of 0.946, still exhibited strong accuracy. However, the slightly inferior performance of LSTM compared to MLP can be attributed to the relatively limited dataset used in this study. Deep learning models like LSTM typically require large amounts of data to optimize their hyperparameters effectively and fully capture complex temporal dependencies. In contrast, MLPs, being shallower architectures, are better suited for small to moderate-sized datasets and are easier to train under these conditions.

Thus, although LSTM models have theoretical advantages in time series modeling, in this specific case, MLP integrated with appropriate feature selection proved more effective, likely due to the scale and characteristics of the available data. Benchmarking the Best Models for Daily PV Output Forecasting is displayed in Table 4. In order to manage electricity supply stability and maximize solar energy production, forecasting is crucial. The performance metrics of several models are shown in this table, emphasizing how well they predict daily PV output.

## Conclusion

This study rigorously evaluated the effectiveness of different machine learning (ML) and deep learning (DL) models for the forecasting of daily photovoltaic (PV) power production. A special focus was placed on the role of feature selection techniques in enhancing predictive performance.

The results clearly demonstrated that *feature selection is a critical step* in the development of reliable forecasting models. By identifying and retaining only the most informative predictors—such as Global Solar Radiation and Number of Hours—feature selection not only improved model accuracy but also reduced computational complexity and mitigated overfitting. For instance, the integration of feature selection techniques like ReliefF with MLP significantly boosted forecasting performance, achieving the lowest MAE (1.6018 kW) and highest R^2^ (0.9608). This highlights how eliminating redundant or irrelevant features leads to more robust and generalizable models.

Furthermore, comparing model performances revealed important insights about *model scalability relative to dataset size*. The MLP model consistently outperformed the LSTM model under the conditions of limited data availability. While deep learning techniques like LSTM are theoretically powerful due to their ability to model temporal dependencies and nonlinearities, their success heavily depends on access to *large, diverse datasets*. In scenarios with *moderate or small datasets*, simpler models such as MLPs, when combined with appropriate feature selection, can not only train faster but also generalize better, yielding superior predictive results.

Additionally, Random Forest, a classical ensemble ML technique, showed strong performance but was slightly outpaced by the MLP integrated with feature selection. This suggests that *combining intelligent feature engineering with suitable model complexity* can often surpass more complex or black-box models, especially when resources and data are limited.

In conclusion, the findings underline three pivotal lessons:

- *Feature selection techniques* are indispensable tools that improve model accuracy, training speed, and interpretation by emphasizing the most influential predictors.
- *Deep learning models require large-scale data* to reach their full potential; otherwise, traditional ML models might deliver better or comparable results with less computational burden.
- *Strategic model selection*, guided by dataset size, feature quality, and forecasting goals, is crucial for building high-performing predictive models in solar energy applications.

Going forward, future work should focus on expanding the dataset (e.g., multi-year measurements from different geographical locations), investigating hybrid models (e.g., feature-engineered LSTM or MLP-ensemble architectures), and exploring automatic feature selection methods to further refine PV power forecasting accuracy.

## Data Availability

The data used and/or analyzed during the current study are available from co-author Dr. Abdelaziz Rabehi (rab_ehi@hotmail.fr) on reasonable request.
